# Anti-Pyretic, Analgesic, and Anti-Inflammatory Activities of Meloxicam and Curcumin Co-Encapsulated PLGA Nanoparticles in Acute Experimental Models

**DOI:** 10.3390/metabo13080935

**Published:** 2023-08-10

**Authors:** Bilal Aslam, Asif Hussain, Muhammad Usman Bari, Muhammad Naeem Faisal, Zia ud Din Sindhu, Rasha Alonaizan, Rasha K. Al-Akeel, Shabana Naz, Rifat Ullah Khan

**Affiliations:** 1 Institute of Physiology and Pharmacology, University of Agriculture Faisalabad, Faisalabad 38040, Pakistan; bilal.aslam@uaf.edu.pk (B.A.); asifhussain072@gmail.com (A.H.); usmanbari22@gmail.com (M.U.B.); m.naeem.faisal@uaf.edu.pk (M.N.F.); 2 Department of Pharmacy, Riphah International University Faisalabad, Faisalabad 38000, Pakistan; 3 Department of Parasitology, University of Agriculture Faisalabad, Faisalabad 38040, Pakistan; sandhu@uaf.edu.pk; 4 Department of Zoology, College of Science, King Saud University, P.O. Box 2455, Riyadh 11451, Saudi Arabia; ralonezan@ksu.edu.sa (R.A.); ralogial@ksu.edu.sa (R.K.A.-A.); 5 Department of Zoology, Government College University, Faisalabad 38000, Pakistan; drshabananaz@gcuf.edu.pk; 6 Faculty of Animal Husbandry and Veterinary Sciences, The University of Agriculture, Peshawar 25130, Pakistan

**Keywords:** meloxicam, curcumin, co-encapsulation, pyrexia, nociception, inflammation

## Abstract

Herein, we evaluated the in vivo effects of meloxicam and curcumin co-encapsulated PLGA nanoparticles in experimental acute models of pyrexia, nociception, and inflammation. Seven groups (*n* = 6) were designed for each investigation and pretreated intraperitoneally (i.p.): the control group, meloxicam (4 mg/kg b.w.), curcumin (15 mg/kg b.w.), and equivalent content containing PLGA capped nanoparticles of meloxicam (Mlx-NP) and curcumin (Cur-NP) alone and in combination (Mlx-Cur-NP; at two doses). The results showed that PLGA encapsulation significantly (*p* ≤ 0.05) improved the in vivo activities of each compound. Furthermore, co-encapsulation of meloxicam and curcumin potentiated the anti-pyretic effect on yeast-induced pyretic rats, anti-nociceptive effect on nociception induced in rats by formalin and heat, and anti-edematogenic activity in xylene-induced ear edema in rats in a dose-dependent manner. In carrageenan-induced paw inflammation in rats, meloxicam and curcumin co-loading (Mlx-Cur-NP) resulted in significant (*p* ≤ 0.05) inhibition of paw inflammation, reduction in TNF-α and PGE2 levels, downregulation of expressions of pro-inflammatory cytokines (TNF-α, IL-1β, and IL-6), as well as a decrease in histopathological changes and TNF-α immunoexpression in paw tissues. Moreover, Mlx-Cur-NP demonstrated noteworthy potentiation in pharmacological effects compared to free compounds and mono-compound-loaded nanoparticles. Thus, the association of meloxicam with curcumin in a biodegradable nanocarrier system could provide a promising anti-pyretic, anti-nociceptive, and anti-inflammatory therapeutic approach for acute conditions.

## 1. Introduction

Inflammation is a non-specific immunological protective response to harmful stimuli, such as chemical substances, infections, or physical injuries. It is consistently seen as a risk factor involved in a number of pathophysiological complications [1]. In its acute stage, an inflammatory reaction supplies the host with an essential defensive response. Numerous pro-inflammatory mediators, including tumor necrosis factor-alpha (TNF-α), interleukins (IL-1β and IL-6), and nitric oxide (NO), are generated at the injury site to provide protection against damaging stimuli [2,3]. These pro-inflammatory mediators play a vital role in ensuring host survival by reversing tissue damage. However, excessive release of these inflammatory mediators may accelerate inflammatory reactions, resulting in significant tissue damage, organ disruptions, and even death [2,4].

Toll-like receptors (TLRs) are immune pattern recognition receptors, which are primarily responsible for sensing and recognizing hazardous signals [5]. TLRs stimulation further activates the transcription factor NF-κB, which induces the generation of several inflammatory mediators, such as TNF-α, IL-1β, and IL-6 [6,7]. The presence of these mediators and cytokines can define the degree of inflammation, and additionally, they can stimulate and sensitize nociceptors [8]. Inflammation causes abrupt pain development, resulting in allodynia and hyperalgesia at the site of injury [9]. Furthermore, the inflammatory mediators tend to trigger the arachidonic acid cascade, which subsequently generates prostaglandin E2 (PGE2), which is the last mediator that induces febrile reactions. Pyrexia occurs when PGE2 binds to its EP3-receptor located in hypothalamus [10].

A variety of potent synthetic and natural compounds have been utilized to treat pain, inflammation, and pyrexia [11,12]. Meloxicam is a non-steroidal anti-inflammatory drug (NSAID), which has analgesic, anti-pyretic, and anti-inflammatory properties. It selectively blocks cyclooxygenase-2 (COX-2) enzyme and prevents prostaglandin (PG) production by inhibiting the metabolism of arachidonic acid. Meloxicam has been shown to be beneficial in the treatment of osteoarthritis, rheumatoid arthritis, and other agonizing conditions, such as cancer surgery, injuries, and dental infections [13]. Animal studies also reported gastric metaplasia, hepatotoxicity, and nephrotoxicity linked to meloxicam [14]. Administration of lower doses or in combination with natural anti-inflammatory compounds, such as resveratrol, curcumin, or omega-3 essential fatty acids, may be an alternative to reduce the side effects of meloxicam [15,16,17]. Curcumin is the main nutraceutical compound extracted from the rhizome of turmeric (*Curcuma longa* L.). Its potent anti-microbial, antioxidant, anti-inflammatory, and anti-tumor properties make it a potential candidate to treat inflammatory conditions linked to oxidative stress [18]. Despite these advantages, both meloxicam and curcumin have poor water solubility and limited bioavailability [19,20].

The utilization of nano-drug delivery systems can help increase the therapeutic efficacy of such agents, which is of prime importance [21]. Designing nanoformulations using polymeric nanoparticles and loading these therapeutic agents into them is one of the potential ways to improve bioavailability, enhance therapeutic efficacy, and decrease adverse effects [22,23]. Additionally, nanoformulations decrease drug toxicity via hindering the drug’s action due to its encapsulation in a polymeric matrix and prolonging its time in the bloodstream [24,25]. Polymeric nanoformulations synthesized from polyesters, such as poly(lactic-co-glycolic) acid (PLGA), gained particular attention because of their non-toxicity, biodegradability, biocompatibility, lower dose needs, and stability in biological fluids and during storage. PLGA has a high drug loading capacity and is frequently used to encapsulate a wide range of therapeutic agents alone or in combination inside the polymer matrix for sustained drug delivery applications [26]. In this context, many efforts have been made to study the improvement of the pharmacological effects of co-encapsulated drug delivery systems. Accordingly, meloxicam and curcumin co-encapsulation improved neuroprotection in a mouse model of Alzheimer’s disease induced by β-amyloid peptide [27]. Furthermore, Della Rocca et al. [28] demonstrated that curcumin co-loading with meloxicam resulted in potentially increased analgesic effect.

In a previous study, we synthesized meloxicam and curcumin co-encapsulated PLGA nanoparticles with the solvent evaporation method and physicochemically characterized them, and it was observed that curcumin co-loading with meloxicam enhanced the antioxidant activity in comparison to nanoparticles loaded with a single compound [29]. The current study aimed to evaluate the anti-pyretic, analgesic, and anti-inflammatory effects of meloxicam and curcumin co-loaded PLGA nanoparticles in experimental animal models of pyrexia, nociception, and inflammation.

## 2. Materials and Methods

### 2.1. Chemicals

Meloxicam (cas#71125-38-7, Sigma-Aldrich^®^, Inc., St. Louis, MO, USA), curcumin (cas#458-37-7, Spectrum^®^, Shanghai, China), poly(lactic-co-glycolic) acid (PLGA) of MW 7000-17,000 (cas#26780-50-7, Sigma-Aldrich^®^, Inc., St. Louis, MO, USA), polyvinyl alcohol (PVA-1500) (cas#9002-89-5, Duksan^®^ Pure Chemicals, Ansan, Republic of Korea), and carrageenan (cat#S18W8685, Sigma-Aldrich^®^, Inc., St. Louis, MO, USA) were purchased.

### 2.2. Preparation and Purification of Nanoparticles

A modified solvent evaporation (oil-in-water (o/w) single-emulsion) technique [30] was used for the synthesis of mono- and dual-compound-loaded PLGA nanoparticles. The acetone solutions of PLGA polymer (200 mg/mL) and each compound (meloxicam and curcumin) dissolved separately in acetone-dichloromethane (1:2 *v*/*v*) mixture were emulsified with 2% (*w*/*v*) aqueous solution of PVA by sonication (output power of 50 W) for 30 s using a micro-tip probe sonicator (Sonics & Materials, Inc., Newtown, CT, USA) in an ice bath. The dual compounds containing nanoparticles were prepared following a similar procedure. All preparations were continuously stirred in a magnetic stirrer at 37 °C for evaporation of the organic phase. After centrifugation at 25,000 rpm (4 °C) for 10 min, the collected pellets were washed with Milli-Q water, lyophilized, and stored at −20 °C.

### 2.3. Experimental Animals

Adult Wistar rats of either sex weighing 160 to 230 g were utilized in this research. Rats were kept under uniform experimental conditions, such as 12 h day/night cycles, 50–60% humidity, and 25 ± 1 °C room temperature. A standard pellet diet and free access to clean drinking water were provided. Before the tests, rats were acclimatized to their surroundings for at least one week. The Institutional Bioethical Committee, Institute of Physiology and Pharmacology, University of Agriculture, Faisalabad, Pakistan (Letter no. 8792/ORIC), approved the research protocols, including animal handling.

### 2.4. Acute Toxicity Test

Rats were grouped (*n* = 3) for the 300 mg/kg b.w. limit test according to OECD-423 guidelines (OECD, 2001). Animals fasted overnight, and treatments, including free compounds (meloxicam and curcumin) and mono-compound-loaded nanoparticles of meloxicam (Mlx-NP) and curcumin (Cur-NP), and co-loaded nanoparticles (Mlx-Cur-NP), were given at a dose rate of 5, 50, and 300 mg/kg b.w. (i.p.) and monitored for 24 h to observe the mortality rate. Various parameters, including behavioral changes, hyperactivity, salivation, respiration, defecation, and urination, were monitored during the first 4 h of treatments. Based on the findings of the toxicity test, the direct limit test was performed. An appropriate dose of treatments was administered daily and kept under surveillance to monitor gross behavioral changes and mortality for 14 days.

### 2.5. Dose Selection for In Vivo Studies

After assessing acute toxicity in rats and the literature review, animal studies were conducted using 4 mg/kg b.w. of meloxicam [31] and 15 mg/kg b.w. of curcumin [32], PLGA capped mono-compound-loaded nanoparticles of meloxicam (Mlx-NP) and curcumin (Cur-NP), and co-loaded nanoparticles (Mlx-Cur-NP) at two doses. All treatments, as given in Table 1, were dissolved in normal saline and administered via intraperitoneal (i.p.) route.

### 2.6. Yeast-Induced Pyrexia Model

The anti-pyretic efficacy was studied using yeast-induced hyperthermia in rats [33]. The rectal temperature of each rat was measured over a 1 h period, and average temperature was calculated. Pyrexia was induced by subcutaneously injecting 10 mL/kg b.w. of 15% yeast aqueous suspension in the back and below the neck of rats. Rats that showed 0.5 °C or above elevation in temperature were selected. Animals were allotted to seven groups (each group comprising six rats) and were administered with free compounds, as well as their encapsulated mono- and dual-compound-loaded nanoparticles (Table 1). After 1 h of treatments, rectal temperatures were re-recorded with a digital thermometer at 1 h interval up to 4 h.

### 2.7. Formalin-Induced Pain Model

The analgesic effect was evaluated by the formalin-induced paw-licking response model of rats [34]. In this regard, treatments including free compounds, mono-compound-loaded nanoparticles, and dual-compound-loaded nanoparticles at low and high doses (Table 1) were administered intraperitoneally in seven groups (*n* = 6) of rats. After 1 h, 50 µL of formalin solution (5% *v*/*v*) was subcutaneously injected into the left hind paw of rats. Paw-licking reflex as pain response of each rat was recorded during early (0–10 min) and late (10–60 min) phases.

### 2.8. Heat-Induced Nociception Model

The hotplate-induced nociception test was performed to assess the analgesic potential of nanoparticles [35]. Animals were screened through a sensitivity test by keeping rats at 52.5 ± 1 °C for 15 s. Rats that showed jumping and paw licking within 15 s were selected for the study and placed into seven groups (*n* = 6). Meloxicam and curcumin in free and encapsulated forms (Table 1) were given intraperitoneally. The response of animals was re-recorded (in sec) at 0.5, 1, 2, and 3 h of treatments administered by placing each rat on a hotplate. A cut-off time of 20 s was implemented to avoid any heat-induced tissue damage.

### 2.9. Xylene-Induced Ear Edema Model

Anti-edematogenic activity was evaluated through the xylene-induced ear edema model of rats [36]. Seven groups of rats (*n* = 6) were pretreated with free compounds and nanoparticles (Table 1). About 30 µL of xylene was injected into the inner surface of the right ear of each rat, while the left ear was kept under control. Rats were executed by cervical dislocation after 2 h of xylene daubing. A 7 mm^2^ cork borer was used to obtain the spherical sections of both ears of each rat to determine the weight difference.

### 2.10. Carrageenan-Induced Paw Inflammation Model

Anti-inflammatory activity was determined using a paw inflammation model of rats induced by carrageenan [37]. Rats were distributed into seven groups (*n* = 6) and given meloxicam, curcumin, Mlx-NP, Cur-NP, and Mlx-Cur-NP at low (L-Mlx-Cur-NP) and high (H-Mlx-Cur-NP) doses (Table 1). After 1 h, 100 μL of 1% carrageenan (suspended in normal saline) was injected into the subplantar tissue of the left hind paw of rats. Paw inflammation was measured in all groups by measuring the diameter of ankle joint with the help of a digital vernier caliper at 0, 0.5, 1, 2, 3, and 4 h after carrageenan injection.

#### 2.10.1. Blood and Organ Sampling

Within 4 h of carrageenan injection, blood samples were collected through a cardiac puncture under the influence of anesthesia (a combination of xylazine 10 mg/kg b.w. and ketamine 90 mg/kg b.w.), incubated for 30 min, and centrifuged at 3000 rpm for 15 min. Separated sera were stored at −20 °C for biochemical analysis. Rats were decapitated, and carrageenan-injected hind paw tissues of rats were properly preserved for qRT-PCR analysis, histological examination, and immunohistochemical analysis.

#### 2.10.2. Determination of TNF-α and PGE2 Levels

Tumor necrosis factor-alpha (TNF-α) and prostaglandin-E2 (PGE2) levels in the sera of control and treated rats were measured using ELISA kits for TNF-α (cat#E0764Ra, BioTech Lab^®^, Beijing, China) and PGE2 (cat#E0504Ra, BioTech Lab^®^, China) with the help of an ELISA reader (Multiskan Go™, Thermo-Scientific, Oxford, UK).

#### 2.10.3. Gene Expression Analysis (qRT-PCR)

Paw tissues stored in RNALater^®^ were homogenized, and total RNA extraction was carried out according to the TRIzol (Thermo-Scientific^®^, UK) method. Total RNA samples were quantified with the help of a Nanodrop spectrophotometer. Reverse transcription was performed using the cDNA synthesis kit (cat#679029, Thermo-Scientific^®^, UK), according to the manufacturer’s instructions. For amplification and quantification, Maxima Syber Green/ROX Master Mix 2X (cat#896415, Thermo-Scientific^®^, UK), nuclease-free water (cat#AM9932, Ambion^®^, Naugatuck, CT, USA), and oligo-primers (Macrogen^®^, Rockville, MD, USA)—TNF-α (F– 5′CACTCTTTCCAGGCCTTTGGG3′, R–5′GCATAGGTCTTCCTGCGGTCA3′), IL-1β (F–5′GCACAGTTCCCCAACTGGTA3′, R–5′GGAGACTGCCCATTCTCGAC3′), and IL-6 (F–5′CATTCTGTCTCGAGCCCACC3′, R–5′TGTGGGTGGTATCCTCTGTGA3′)—were utilized, and analysis was conducted using a thermal cycler (iQ5 Bio-Rad). Finally, the qRT-PCR data were analyzed using the 2^−ΔΔCt^ method, keeping β-actin as a reference gene to determine relative mRNA expressions.

#### 2.10.4. Histopathological Examination

Paw tissues preserved in 10% formalin buffer were embedded in paraffin, and tissue sections of 5–6 μm thickness were prepared using a microtome. Sections were stained with H&E dyes. The prepared slides were observed under a light microscope (IRMECO^®^, Lutjensee, Germany) for pathological changes. Histopathological changes, including infiltration of inflammatory cells and cartilage damage, were assessed semi-quantitatively, according to a previously described scoring (0–4) method [38]. Score 0: absence of degenerative changes (<1%), score 1: mild changes (1–25%), score 2: mild to moderate changes (26–50%), score 3: moderate to severe changes (51–75%), and score 4: severe degenerative changes (>75%).

#### 2.10.5. Immunohistochemistry Analysis

For immunohistochemistry, deparaffinized and hydrated paw tissue sections (4–5 μm of thickness) were immersed in 0.1 M citrate buffer (pH 6) and heated at 95 °C for 5 min in the microwave for antigen retrieval. Sections were washed with phosphate-buffered saline (PBS, pH 7.4) and treated with a 3% H_2_O_2_ solution for 15 min to block endogenous peroxidases. Then, the sections were incubated overnight at 4 °C with mouse anti-TNF-α monoclonal primary antibody (1:100 dilution in 5% BSA-PBS, Elabscience^®^, Houston, TX, USA). On the next day, polyperoxidase-anti-mouse/rabbit secondary antibody (anti-IgG, Elabscience^®^, USA) was applied for 20 min at 37 °C. After washing with PBS, sections were stained with 3,3′diaminobenzidine-peroxide (DAB) chromophore, counterstained with Mayer hematoxylin, dehydrated, and examined for TNF-α expression. The IHC score of TNF-α expression in paw tissue was semi-quantitatively analyzed in two random fields per section.

### 2.11. Statistical Analysis

GraphPad Prism^®^ v6.01 (GraphPad, Software Inc., San Diego, CA, USA) was used to statistically analyze the obtained data, and the findings were presented as mean ± standard error of mean (SEM). One-way/two-way ANOVA followed by Tukey’s test was applied to find statistical significance (*p* ≤ 0.05) among the control and treated groups.

## 3. Results

### 3.1. Toxicity Profile of Nanoparticles

The findings of the acute toxicity experiment indicated no adverse effects in rats administered with free compounds (meloxicam and curcumin) and PLGA nanoparticles encapsulating single and dual compounds. The animals did not show any sign of changes in gross appearance or behavior. In addition, no mortality was observed over 14 days.

### 3.2. Effect on Yeast-Induced Hyperthermia in Rats

**Figure 1:**
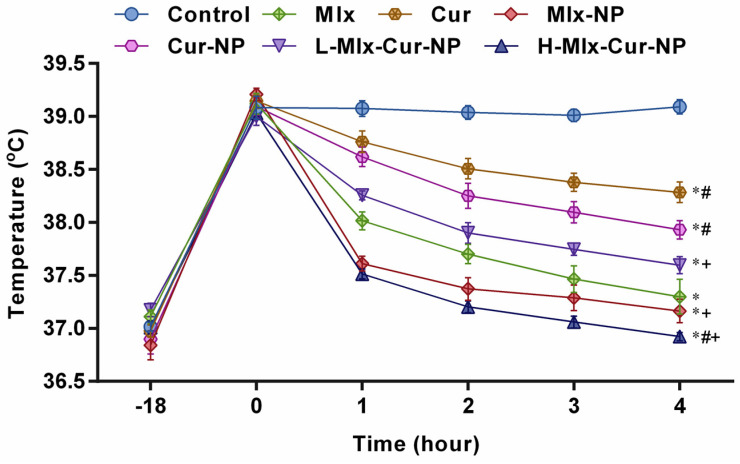
Anti-pyretic effect of meloxicam and curcumin co-encapsulated nanoparticles on yeast-induced pyrexia in rats. Results are presented as mean ± SEM, *n* = 6. * *p* ≤ 0.05, ^#^ *p* ≤ 0.05, ^+^ *p* ≤ 0.05: significance from Control, Mlx, and Cur groups, respectively. Mlx: meloxicam (4 mg/kg b.w.), Cur: curcumin (15 mg/kg b.w.), Mlx-NP: meloxicam-loaded nanoparticles (4 mg/kg b.w.), Cur-NP: curcumin-loaded nanoparticles (15 mg/kg b.w.), L-Mlx-Cur-NP: meloxicam (2 mg/kg b.w.) + curcumin (7.5 mg/kg b.w.) co-loaded nanoparticles, H-Mlx-Cur-NP: meloxicam (4 mg/kg b.w.) + curcumin (15 mg/kg b.w.) co-loaded nanoparticles.

The results displayed in Figure 1 indicate that injection of the yeast suspension markedly increased the rectal temperature, which peaked at 4 h (39.09 ± 0.07 °C). Administration of free meloxicam and curcumin and their mono- and co-loaded nanoparticles significantly (*p* ≤ 0.05) lowered rectal temperatures up to 4 h in comparison with the control group. Treatment with Mlx-NP and H-Mlx-Cur-NP markedly decreased the temperatures after 1 h and continued to decrease them up to 4 h, i.e., 37.16 ± 0.11 °C and 36.92 ± 0.04 °C, respectively. Up to 2 h, free meloxicam and L-Mlx-Cur-NP demonstrated fever attenuation and indicated a marginally significant (*p* ≤ 0.05) change after 3 h following 4 h (meloxicam, 37.30 ± 0.16 °C; L-Mlx-Cur-NP, 37.59 ± 0.08 °C) of treatments as compared to the control group. Meanwhile, free curcumin (38.28 ± 0.09 °C) and Cur-NP (37.93 ± 0.09 °C) showed comparatively less change in hyperthermia up to 4 h of the experiment.

### 3.3. Effect on Formalin-Induced Pain in Rats

**Figure 2:**
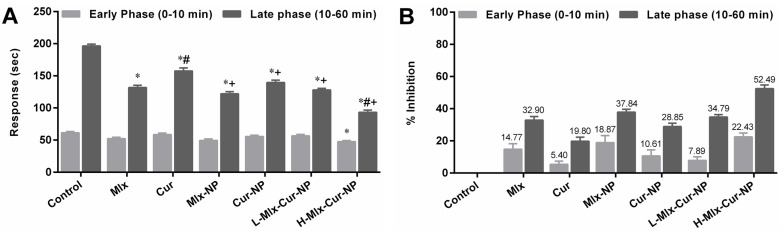
Analgesic effect of meloxicam and curcumin co-loaded nanoparticles on formalin-induced nociception in rats. (**A**) Nociception response (sec) and (**B**) percentage inhibition of nociception. Results are presented as mean ± SEM, *n* = 6. * *p* ≤ 0.05, ^#^ *p* ≤ 0.05, ^+^ *p* ≤ 0.05: significance from Control, Mlx, and Cur groups, respectively. Mlx: meloxicam (4 mg/kg b.w.), Cur: curcumin (15 mg/kg b.w.), Mlx-NP: meloxicam-loaded nanoparticles (4 mg/kg b.w.), Cur-NP: curcumin-loaded nanoparticles (15 mg/kg b.w.), L-Mlx-Cur-NP: meloxicam (2 mg/kg b.w.) + curcumin (7.5 mg/kg b.w.) co-loaded nanoparticles, H-Mlx-Cur-NP: meloxicam (4 mg/kg b.w.) + curcumin (15 mg/kg b.w.) co-loaded nanoparticles.

In the nociception model of rats induced by formalin, the treatments, except H-Mlx-Cur-NP (22.43%), demonstrated non-significant (*p* ≥ 0.05) analgesic effects in contrast to the control group during the early phase of the experiment (Figure 2A,B). In the later phase, Mlx-NP, Cur-NP, L-Mlx-Cur-NP, and H-Mlx-Cur-NP showed significant (*p* ≤ 0.05) analgesic effects in comparison to the control group. In addition, co-encapsulation of meloxicam and curcumin in a dose-dependent manner inhibited pain response in rats, i.e., 34.79% and 52.49% in L-Mlx-Cur-NP and H-Mlx-Cur-NP, respectively. Furthermore, Mlx-NP (37.84%) and Cur-NP (28.85%) showed enhanced analgesic effects as compared to free meloxicam (32.90%) and curcumin (19.80%).

### 3.4. Effect on Heat-Induced Nociception in Rats

**Figure 3:**
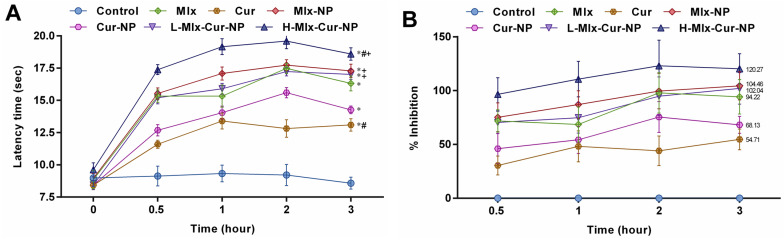
Analgesic effect of PLGA nanoparticles co-encapsulating meloxicam and curcumin against heat-induced pain in rats. (**A**) Latency time (sec) and (**B**) percentage inhibition of nociception. Results are presented as mean ± SEM, *n* = 6. * *p* ≤ 0.05, ^#^ *p* ≤ 0.05, ^+^ *p* ≤ 0.05: significance from Control, Mlx, and Cur groups, respectively. Mlx: meloxicam (4 mg/kg b.w.), Cur: curcumin (15 mg/kg b.w.), Mlx-NP: meloxicam-loaded nanoparticles (4 mg/kg b.w.), Cur-NP: curcumin-loaded nanoparticles (15 mg/kg b.w.), L-Mlx-Cur-NP: meloxicam (2 mg/kg b.w.) + curcumin (7.5 mg/kg b.w.) co-loaded nanoparticles, H-Mlx-Cur-NP: meloxicam (4 mg/kg b.w.) + curcumin (15 mg/kg b.w.) co-loaded nanoparticles.

As shown in Figure 3A,B, PLGA encapsulation of meloxicam and curcumin alone and in combination exhibited significant (*p* ≤ 0.05) improvement in analgesic activity against heat-induced pain in rats compared to the control group. After 0.5 h, H-Mlx-Cur-NP revealed the highest inhibition (96.58%), and it continually showed inhibition up to 3 h (120.27%). Meanwhile, free meloxicam (71.84%), Mlx-NP (75.24%), and L-Mlx-Cur-NP (70.47%) demonstrated pain inhibition after 0.5 h and the maximum analgesic effect observed at 3 h (94.22%, 104.46%, and 102.04%, respectively). However, Cur-NP showed 68.13% inhibition at 3 h, which was higher than the 54.71% inhibitory effect of free curcumin against external pain stimulus.

### 3.5. Effect on Xylene-Induced Ear Edema in Rats

**Figure 4:**
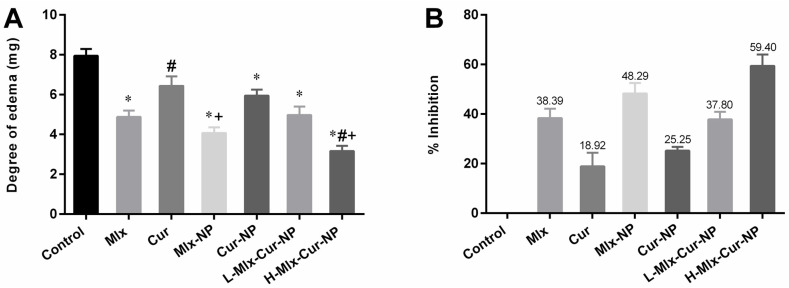
Anti-inflammatory effect of dual-compound-loaded nanoparticles of meloxicam and curcumin on xylene-induced ear edema in rats. (**A**) Degree of ear edema (mg) and (**B**) percentage inhibition of ear edema. Results are displayed as mean ± SEM, *n* = 6. * *p* ≤ 0.05, ^#^ *p* ≤ 0.05, ^+^ *p* ≤ 0.05: significance from Control, Mlx, and Cur groups, respectively. Mlx: meloxicam (4 mg/kg b.w.), Cur: curcumin (15 mg/kg b.w.), Mlx-NP: meloxicam-loaded nanoparticles (4 mg/kg b.w.), Cur-NP: curcumin-loaded nanoparticles (15 mg/kg b.w.), L-Mlx-Cur-NP: meloxicam (2 mg/kg b.w.) + curcumin (7.5 mg/kg b.w.) co-loaded nanoparticles, H-Mlx-Cur-NP: meloxicam (4 mg/kg b.w.) + curcumin (15 mg/kg b.w.) co-loaded nanoparticles.

In the ear edema model of rats, considerable ear edema was noticed in the control group after 2 h of xylene injection (Figure 4A,B). In comparison to the control group, PLGA encapsulated mono- and dual-compound-loaded nanoparticles, including Mlx-NP, Cur-NP, L-Mlx-Cur-NP, and H-Mlx-Cur-NP, showed significant (*p* ≤ 0.05) ear edema inhibition compared to free meloxicam and curcumin. Administration of L-Mlx-Cur-NP (37.80%) and H-Mlx-Cur-NP (59.40%) exhibited dose-dependent and enhanced anti-inflammatory effects in contrast to Mlx-NP (48.29%) and Cur-NP (25.25%).

### 3.6. Effect on Carrageenan-Induced Paw Inflammation in Rats

**Figure 5:**
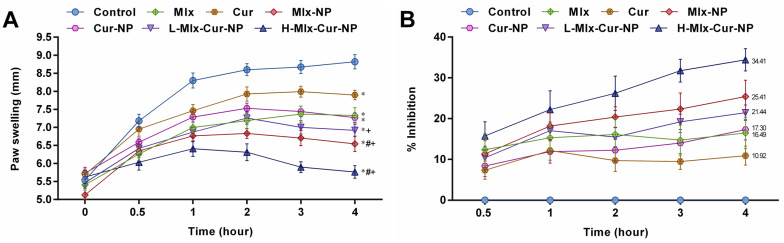
Anti-inflammatory effect of dual-compound-loaded nanoparticles on paw inflammation in rats induced by carrageenan. (**A**) Changes in paw inflammation (mm) and (**B**) percentage inhibition of paw inflammation. Results are presented as mean ± SEM, *n* = 6. * *p* ≤ 0.05, ^#^ *p* ≤ 0.05, ^+^ *p* ≤ 0.05: significance from Control, Mlx, and Cur groups, respectively. Mlx: meloxicam (4 mg/kg b.w.), Cur: curcumin (15 mg/kg b.w.), Mlx-NP: meloxicam-loaded nanoparticles (4 mg/kg b.w.), Cur-NP: curcumin-loaded nanoparticles (15 mg/kg b.w.), L-Mlx-Cur-NP: meloxicam (2 mg/kg b.w.) + curcumin (7.5 mg/kg b.w.) co-loaded nanoparticles, H-Mlx-Cur-NP: meloxicam (4 mg/kg b.w.) + curcumin (15 mg/kg b.w.) co-loaded nanoparticles.

PLGA-capped mono- and dual-compound-loaded nanoparticles of meloxicam and curcumin demonstrated improvement in anti-inflammatory activity through inhibition of carrageenan-induced paw inflammation in rats (Figure 5A,B). The results showed that carrageenan injection induced significant paw inflammation. Additionally, it was observed that the anti-inflammatory activities of free meloxicam (16.49%) and curcumin (10.92%) were significantly improved when given in the encapsulated form, i.e., Mlx-NP (25.41%) and Cur-NP (17.30%). Rats treated with co-loaded nanoparticles at low dose (L-Mlx-Cur-NP, 21.44%) and high dose (H-Mlx-Cur-NP, 34.41%) revealed dose-dependent inflammation inhibition up to 4 h. Among single- and dual-compound encapsulated nanoparticles, H-Mlx-Cur-NP showed a noteworthy anti-inflammatory effect after 0.5 h to 4 h of the study.

#### 3.6.1. Effect on TNF-α and PGE2 Levels

**Figure 6:**
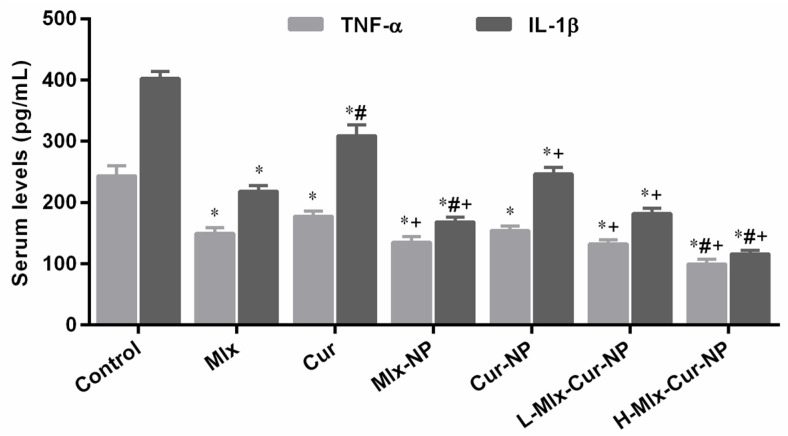
Effect of free meloxicam and curcumin mono- and dual-compound-loaded nanoparticles on tumor necrosis factor-α (TNF-α) and prostaglandin E2 (PGE2) levels in sera of carrageenan-injected rats. Data are presented as mean ± SEM, *n* = 6. * *p* ≤ 0.05, ^#^ *p* ≤ 0.05, ^+^ *p* ≤ 0.05: significance from Control, Mlx, and Cur groups, respectively. Mlx: meloxicam (4 mg/kg b.w.), Cur: curcumin (15 mg/kg b.w.), Mlx-NP: meloxicam-loaded nanoparticles (4 mg/kg b.w.), Cur-NP: curcumin-loaded nanoparticles (15 mg/kg b.w.), L-Mlx-Cur-NP: meloxicam (2 mg/kg b.w.) + curcumin (7.5 mg/kg b.w.) co-loaded nanoparticles, H-Mlx-Cur-NP: meloxicam (4 mg/kg b.w.) + curcumin (15 mg/kg b.w.) co-loaded nanoparticles.

Figure 6 demonstrates that carrageenan administration resulted in a substantial increase in TNF-α and PGE2 levels in the sera of rats. Treatment with free compounds and their encapsulated mono- and co-encapsulated nanoparticles significantly (*p* ≤ 0.05) reduced the levels of TNF-α and PGE2 in comparison to the control group. A significant reduction was observed in the TNF-α and PGE2 levels of Mlx-NP, Cur-NP, L-Mlx-Cur-NP, and H-Mlx-Cur-NP treatment groups in comparison to the free meloxicam and curcumin treatment groups, while a dose-dependent decrease in TNF-α and PGE2 levels was noticed in the L-Mlx-Cur-NP and H-Mlx-Cur-NP treatment groups. Additionally, H-Mlx-Cur-NP exhibited the highest effect among all treatments. However, curcumin in the free and encapsulated form (Cur-NP) showed marginally significant effects on these parameters as compared to other treatments.

#### 3.6.2. Effect on Expression Levels of Inflammatory Cytokines

**Figure 7:**
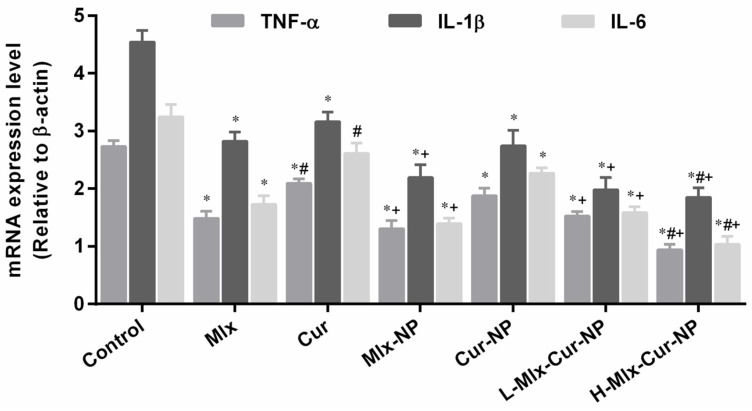
Modulatory effects of mono- and co-encapsulated nanoparticles on mRNA expression levels of tumor necrosis factor-α (TNF-α), interleukin-1β (IL-1β), and interleukin-6 (IL-6) in paw tissues. Data are presented as mean ± SEM, *n* = 6. * *p* ≤ 0.05, ^#^ *p* ≤ 0.05, ^+^ *p* ≤ 0.05: significance from Control, Mlx, and Cur groups, respectively. Mlx: meloxicam (4 mg/kg b.w.), Cur: curcumin (15 mg/kg b.w.), Mlx-NP: meloxicam-loaded nanoparticles (4 mg/kg b.w.), Cur-NP: curcumin-loaded nanoparticles (15 mg/kg b.w.), L-Mlx-Cur-NP: meloxicam (2 mg/kg b.w.) + curcumin (7.5 mg/kg b.w.) co-loaded nanoparticles, H-Mlx-Cur-NP: meloxicam (4 mg/kg b.w.) + curcumin (15 mg/kg b.w.) co-loaded nanoparticles.

To elucidate the molecular mechanism underlying the anti-inflammatory effect of mono- and co-loaded nanoparticles, the mRNA expression levels of pro-inflammatory cytokines (TNF-α, IL-1β, and IL-6) in paw tissue were determined (Figure 7). Carrageenan induced a significant upregulation of TNF-α, IL-1β, and IL-6 expressions. In comparison to the control group, PLGA encapsulation of meloxicam and curcumin alone (Mlx-NP and Cur-NP) or in combination (L-Mlx-Cur-NP and H-Mlx-Cur-NP) significantly (*p* ≤ 0.05) decreased the expressions of pro-inflammatory cytokines (TNF-α, IL-1β, and IL-6). In particular, Mlx-Cur-NP exhibited dose-dependent effects on inflammatory cytokines, and H-Mlx-Cur-NP showed the highest anti-inflammatory effect among all treatments.

#### 3.6.3. Histopathological Findings

**Figure 8:**
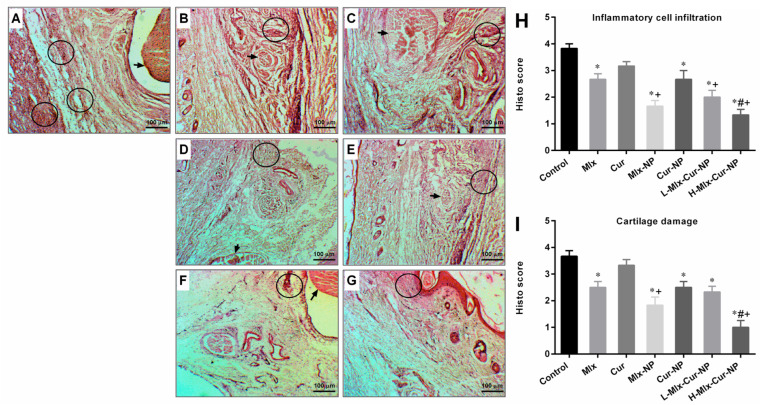
H&E stained photomicrographs of carrageenan-injected hind paws of rats (magnification ×100, scale bar 100 µm). (**A**) Control group showed a marked infiltration of inflammatory cells, granulation and edema of tissue, and cartilage damage (circle: inflammatory cell infiltration, arrow: cartilage damage). (**B**,**C**) Carrageenan model rats given free meloxicam and curcumin indicated a moderate degree of degenerative changes. (**D**,**E**) Mlx-NP and Cur-NP groups demonstrated relatively improved paw histology with less inflammatory reaction. (**F**,**G**) L-Mlx-Cur-NP and H-Mlx-Cur-NP groups exhibited a dose-related improvement of tissue inflammatory reaction and cartilage abrasion. Meanwhile, H-Mlx-Cur-NP treated rats showed better histological features among all groups. Semi-quantitative scores of (**H**) infiltration of inflammatory cells and (**I**) cartilage damage are presented as mean ± SEM, *n* = 6. * *p* ≤ 0.05, ^#^ *p* ≤ 0.05, ^+^ *p* ≤ 0.05: significance from Control, Mlx, and Cur groups, respectively. Mlx: meloxicam (4 mg/kg b.w.), Cur: curcumin (15 mg/kg b.w.), Mlx-NP: meloxicam-loaded nanoparticles (4 mg/kg b.w.), Cur-NP: curcumin-loaded nanoparticles (15 mg/kg b.w.), L-Mlx-Cur-NP: meloxicam (2 mg/kg b.w.) + curcumin (7.5 mg/kg b.w.) co-loaded nanoparticles, H-Mlx-Cur-NP: meloxicam (4 mg/kg b.w.) + curcumin (15 mg/kg b.w.) co-loaded nanoparticles.

Histopathological observations indicated a marked inflammatory cell infiltration and destruction of cartilage in rats’ hind paw tissue of carrageenan control. H&E stained histo-images and scores of inflammatory cells and cartilage damage are presented in Figure 8. Free meloxicam and curcumin groups moderately reduced these inflammatory process indications; however, curcumin did not show significant difference from the control group. Mlx-NP, Cur-NP, L-Mlx-Cur-NP, and H-Mlx-Cur-NP groups demonstrated a significant improvement in paw histology in comparison to free meloxicam or curcumin administered groups. Additionally, H-Mlx-Cur-NP promisingly attenuated histological changes in rats’ paws compared to Mlx-NP, Cur-NP, and L-Mlx-Cur-NP.

#### 3.6.4. Immunohistochemistry Findings

**Figure 9:**
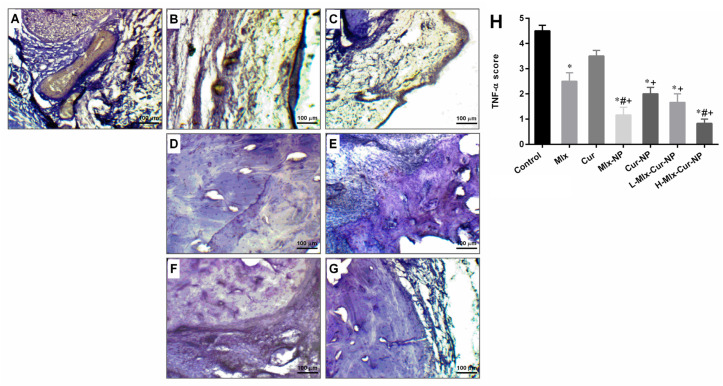
Immunohistochemical stained sections of rats’ hind paw for TNF-α expression (magnification ×100, scale bar 100 µm). (**A**) Carrageenan control rats showed intense expression of TNF-α. (**B**,**C**) Rats from free meloxicam and curcumin groups displayed relatively decreased TNF-α expression in paw tissues. (**D**–**G**) Mlx-NP, Cur-NP, L-Mlx-Cur-NP, and H-Mlx-Cur-NP groups showed a mild expression of TNF-α. (**H**) Semi-quantitative IHC score of TNF-α expression. Values are presented as mean ± SEM, *n* = 6. * *p* ≤ 0.05, ^#^ *p* ≤ 0.05, ^+^ *p* ≤ 0.05: significance from Control, Mlx, and Cur groups, respectively. Mlx: meloxicam (4 mg/kg b.w.), Cur: curcumin (15 mg/kg b.w.), Mlx-NP: meloxicam-loaded nanoparticles (4 mg/kg b.w.), Cur-NP: curcumin-loaded nanoparticles (15 mg/kg b.w.), L-Mlx-Cur-NP: meloxicam (2 mg/kg b.w.) + curcumin (7.5 mg/kg b.w.) co-loaded nanoparticles, H-Mlx-Cur-NP: meloxicam (4 mg/kg b.w.) + curcumin (15 mg/kg b.w.) co-loaded nanoparticles.

The paw tissue of rats in the carrageenan control group showed intensive expression of TNF-α, as the IHC images and TNF-α expression scores in Figure 9 show. The treatment groups indicated variable degrees of reduction in TNF-α expression. Treatment with free meloxicam revealed reduction in the expression of TNF-α, while treatment with mono- and dual-compound-loaded nanoparticles, including Mlx-NP, Cur-NP, L-Mlx-Cur-NP, and H-Mlx-Cur-NP, resulted in a significant decrease in TNF-α expression. Furthermore, H-Mlx-Cur-NP administration resulted in comparatively very mild expression of the inflammatory marker.

## 4. Discussion

The findings of this study indicated that polymeric co-encapsulation of meloxicam and curcumin (Mlx-Cur-NP) potentiated anti-pyretic, anti-nociceptive, and anti-inflammatory responses, as demonstrated in the different acute animal models of pyrexia, nociception, and inflammation, compared to free compounds (meloxicam and curcumin) and individual compound-loaded nanoparticles (Mlx-NP and Cur-NP).

The hypothalamus plays a key role in the regulation of normal body temperature, as it is required for normal functioning and cellular metabolic activities. Various endogenous mediators, such as PGE2, TNF-α, IL-6, IL-8, IL-1β, and macrophage protein-1, are involved in fever induction [39]. The anti-pyretic activities of Mlx-Cur-NP in comparison to free compounds and mono-compound-loaded nanoparticles, i.e., Mlx-NP and Cur-NP, were studied in yeast-induced pyretic rats. A significant dose-dependent decrease in temperature was noted with Mlx-Cur-NP as compared with Mlx-NP and Cur-NP, as well as free meloxicam and curcumin. Among the intraperitoneal treatments administered, the H-Mlx-Cur-NP group exhibited the highest anti-pyretic activity (36.92 ± 0.04 °C), while the temperatures noted in the Mlx-NP, Cur-NP, and L-Mlx-Cur-NP groups were 37.16 ± 0.11 °C, 37.93 ± 0.09 °C, and 37.59 ± 0.08 °C, respectively. Meanwhile, temperatures of 37.30 ± 0.16 °C and 38.28 ± 0.09 °C were recorded in the free meloxicam and curcumin treatment groups at the end of the experiment (Figure 1). These results confirmed the enhancement in biological activities of synthesized nanoparticles and potentiated anti-pyretic activity of co-encapsulated nanoparticles.

Pain is a response to an internal or external stimulus, which could be due to chemical, mechanical, or thermal injury [40]. In the present study, the anti-nociceptive activities were evaluated in nociception models of formalin and heat. In the formalin-induced nociception model, persistent pain was observed in the control group during both phases of the experiment. In the initial phase (neurogenic phase), formalin stimulates the release of bradykinins and tachykinins, mediating the activation of primary afferent fibers and nociceptors. The development of tissue injury in the later phase (inflammatory phase) involves the release of serotonin, histamine, prostaglandins, and other excitatory amino acids [41]. The results of the present study (Figure 2) indicated that only Mlx-Cur-NP at a high dose (H-Mlx-Cur-NP) showed a significant (*p* ≤ 0.05) reduction in pain during the early phase. In the later phase, the induction of nociception due to formalin was significantly (*p* ≤ 0.05) inhibited with meloxicam and curcumin co-encapsulated nanoparticles (Mlx-Cur-NP) administered at a low dose (L-Mlx-Cur-NP: 34.79%) and high dose (H-Mlx-Cur-NP: 52.49%). The treatment with Mlx-NP (37.84%) and Cur-NP (28.85%) exhibited significant pain inhibition as compared to free meloxicam (32.90%) and curcumin (19.80%). The heat-induced nociception model provides valuable findings for the central analgesic effect of test agents against partial tissue damage and sensitivity to potential analgesic compounds [42]. Our study demonstrated that PLGA encapsulation of meloxicam and curcumin significantly (*p* ≤ 0.05) improved analgesic activity, as observed in Mlx-NP, Cur-NP, and Mlx-Cur-NP in comparison to free meloxicam and curcumin (Figure 3). All treatments showed significant (*p* ≤ 0.05) analgesia against nociception stimulus. The highest analgesic activities were observed at 3 h of the experiment. At 3 h, the pain inhibition noted in free meloxicam (94.22%) and curcumin (54.71%) was substantially enhanced in Mlx-NP (104.46%) and Cur-NP (68.13%) groups. Meanwhile, Mlx-Cur-NP demonstrated significant pain inhibition in a dose-dependent manner, as noticed in the L-Mlx-Cur-NP (102.04%) and H-Mlx-Cur-NP (120.27%) treatment groups. 

Xylene-induced ear edema is one of the commonly used models to evaluate anti-inflammatory activity. Xylene injection into the ear is considered to provoke substance-P-mediated neurogenic edema. Substance P is a peptide related to the central and peripheral nervous systems. Its release in sensory neurons along with other neuropeptides stimulates leukocytes, mast cells, and endothelial cells to secrete prostaglandins, histamine, serotonin, cytokines, and nitric oxide. This results in increased vasodilation and plasma extravasation, which subsequently induces neurogenic inflammation and formation of ear edema [43,44]. The present study revealed that xylene-injection-induced ear edema was significantly (*p* ≤ 0.05) decreased with Mlx-NP (48.29%) and Cur-NP (25.25%) in contrast to free meloxicam (38.39%) and curcumin (18.92%). Moreover, administration of Mlx-Cur-NP in a dose-dependent manner inhibited ear edema in rats treated with L-Mlx-Cur-NP (37.80%) and H-Mlx-Cur-NP (59.40%). Notably, Mlx-Cur-NP showed better edema inhibition than mono-compound-loaded nanoparticles (Figure 4).

Carrageenan-induced inflammation is a bi-phasic process, which involves leukocyte infiltration and increased capillary permeability at the injection site. During the initial phase of edema, the release of histamine, serotonin, bradykinins, and prostaglandins (PGE2) occurs, while infiltration of neutrophils and continuous release of prostaglandins and pro-inflammatory cytokines (TNF-α, IL-1β, and IL-6) in paw tissues has been established during the later phase [45]. Local infiltration of neutrophils contributes to inflammation development by generating superoxide and hydroxyl radicals. Additionally, the production of another inflammatory mediator, i.e., nitric oxide (NO) from the independent NO synthase isoform, is believed to be responsible for the amplification of blood flow, vascular permeability, and enhanced pro-inflammatory cytokines’ expression in the inflamed area [46]. TNF-α is an inflammatory cytokine, which promotes leukocyte adherence to the epithelium through the expression of adhesion molecules, as well as vasodilation and edema production. IL-1β is a pro-inflammatory cytokine, which plays critical roles in acute and chronic inflammatory pathologies. Administration of inflammatory substances, such as carrageenan, causes mechanical or thermal hyperalgesia in the inflamed tissue via inducing pro-inflammatory cytokines, including IL-1β and IL-6. It was also proposed that inhibiting IL-1β and IL-6 might be a broad-acting and effective way of treating pain and inflammation [45,47,48]. This study is the first study investigating the anti-inflammatory activity of meloxicam and curcumin co-encapsulated nanoparticles (Mlx-Cur-NP) in carrageenan-induced paw edema in rats. The results showed that carrageenan induced significant paw inflammation (maximum at 4 h of the experiment) (Figure 5), elevation in TNF-α and PGE2 levels in the sera (Figure 6), and upregulation of TNF-α, IL-1β, and IL-6 expressions in paw tissue (Figure 7). Histological examination showed significant infiltration of inflammatory cells and cartilage damage in paw tissue of the carrageenan-injected control group (Figure 8). In addition, TNF-α immunoexpression was found to be markedly profound in paw tissues of control rats (Figure 9). This paw inflammation was inhibited in a dose-dependent manner with Mlx-Cur-NP. The Mlx-Cur-NP treatment significantly reduced the serum levels of TNF-α and PGE2, as well as the mRNA expression levels of TNF-α, IL-1β, and IL-6, and histopathological changes along with TNF-α immunoexpression in paw tissues in a dose-dependent manner. Our findings also suggested that curcumin potentiated the anti-inflammatory effect of meloxicam co-encapsulated nanoparticles (Mlx-Cur-NP) compared to nanoparticles encapsulating meloxicam (Mlx-NP) and/or curcumin (Cur-NP) alone.

The use of biocompatible water-based polymeric nanoparticles was Formulated with the primary objective of mitigating the loss of active compounds and degradation of therapeutic agents, these innovations are designed to amplify drug bioavailability while mitigating undesirable side effects. This is achieved through the augmentation of drug accumulation specifically within targeted tissues and organs [49]. Simultaneous encapsulation of drugs can synergistically enhance the advantageous impacts of active compounds. This co-encapsulation method not only heightens metabolic stability but also provides resilience against enzymatic degradation, ultimately resulting in diminished toxicity [29,50]. The efficacy of curcumin nanoparticles has already been substantiated in animal models, showcasing enhanced anti-inflammatory effects across various conditions such as pyrexia, nociception, and both acute and chronic inflammations. It is suggested that reducing curcumin’s particle size to the nanoscale enhanced its stability. Therefore, much of the active compounds reach the tissue and potentiate meloxicam’s pharmacological properties [27,28,51]. Co-encapsulation of curcumin with another drug is a strategy, which has already shown promising results in other models. Curcumin co-loading with fish oil exhibited neuroprotection via enhancing antioxidant activity in human SH-SY5Y cells [52]. In an experimental study conducted by Coradini et al., curcumin and resveratrol co-loading provided more potent anti-arthritic activity without altering the serum levels of liver function biomarkers [53]. Jain et al. reported that the conjugation of curcumin and diclofenac improved the bioavailability of both agents and markedly attenuated arthritis compared to monotherapy in an arthritis model induced by streptococcal cell wall [54].

## 5. Conclusions

In summary, our findings suggest that curcumin potentiated the pharmacological activities of meloxicam co-encapsulating PLGA nanoparticles in acute animal models. Moreover, nanoencapsulation improved the biological activities of meloxicam and curcumin in monotherapy; however, dual-compound-loaded nanoparticles significantly potentiated the anti-pyretic, anti-nociceptive, and anti-edematogenic activities. Furthermore, meloxicam co-encapsulation with curcumin resulted in a better anti-inflammatory activity in carrageenan-induced paw inflammation than monotherapy. Thus, it might be a potential therapeutic approach to treat pyrexia, nociception, and acute inflammation at lower doses and minimize the associated side effects.

## Figures and Tables

**Table 1:** Treatment protocols for in vivo studies.

Groups	Treatments
GI: Control	Normal saline at 3 mL/kg b.w., i.p.
GII: Meloxicam	Free meloxicam at 4 mg/kg b.w., i.p.
GIII: Curcumin	Free curcumin at 15 mg/kg b.w., i.p.
GIV: Mlx-NP	Meloxicam-loaded nanoparticles (4 mg/kg b.w.), i.p.
GV: Cur-NP	Curcumin-loaded nanoparticles (15 mg/kg b.w.), i.p.
GVI: L-Mlx-Cur-NP	Nanoparticles co-encapsulating meloxicam (2 mg/kg b.w.) + curcumin (7.5 mg/kg b.w.), i.p.
GVII: H-Mlx-Cur-NP	Nanoparticles co-encapsulating meloxicam (4 mg/kg b.w.) + curcumin (15 mg/kg b.w.), i.p.

## Data Availability

The data presented in this study are available in article.
